# The Effects of Financial Education on Impulsive Decision Making

**DOI:** 10.1371/journal.pone.0159561

**Published:** 2016-07-21

**Authors:** William B. DeHart, Jonathan E. Friedel, Jean M. Lown, Amy L. Odum

**Affiliations:** 1 Department of Psychology, Utah State University, Logan, Utah, United States of America; 2 Department of Family, Consumer and Human Development, Utah State University, Logan, Utah, United States of America; Institutes for Behavior Resources and Johns Hopkins University School of Medicine, UNITED STATES

## Abstract

Delay discounting, as a behavioral measure of impulsive choice, is strongly related to substance abuse and other risky behaviors. Therefore, effective techniques that alter delay discounting are of great interest. We explored the ability of a semester long financial education course to change delay discounting. Participants were recruited from a financial education course (n = 237) and an abnormal psychology course (n = 80). Both groups completed a delay-discounting task for $100 during the first two weeks (Time 1) of the semester as well as during the last two weeks (Time 2) of the semester. Participants also completed a personality inventory and financial risk tolerance scale both times and a delay-discounting task for $1,000 during Time 2. Delay discounting decreased in the financial education group at the end of the semester whereas there was no change in delay discounting in the abnormal psychology group. Financial education may be an effective method for reducing delay discounting.

## Introduction

Despite efforts to increase financial literacy, many Americans continue to make shortsighted, impulsive financial choices that ultimately cause harm to their future financial stability [1]. In other words, people frequently make short-term financial decisions that provide more immediate benefits instead of making decisions that are consistent with their long-term financial goals. Many of these types of financial choices can be framed as a trade-off between an immediate, smaller outcome and a larger, delayed outcome, such as saving for retirement, selecting a mortgage or choosing to carry a credit card balance which results in a smaller monthly payment but also accumulates interest. Therefore, the individual is choosing between paying less now but paying more overall or paying more now but less overall. Poor financial choices like overspending can have high personal and societal costs including a greater dependence on family and governmental assistance.

Impulsive financial choices, like other forms of impulsive choice, can be described by delay discounting. Delay discounting is the decrease in value of an outcome as the delay to receiving that outcome increases [2,3]. In delay-discounting tasks, participants choose between smaller, immediate outcomes and larger, delayed outcomes [4,5]. The amount of the immediate choice alternative is adjusted across successive trials to find an indifference point. Indifference points are the amount at which the smaller, immediate and larger, delayed reward have the same value and therefore a person is equally likely to choose either outcome. This adjusting procedure is repeated over a range of delays to the larger later reward to determine a discounting curve [3]. Steeper discounting curves (in which rewards lose value more rapidly with delay) indicate higher degrees of impulsive choice.

Delay discounting curves are described by hyperbolic [2] or hyperbolic-like [6,7] functions, which predict that value decreases proportionally more at shorter delays and proportionally less at longer delays [8]. Importantly, impulsive choice, quantified by hyperbolic models of delay discounting, account for the tendency of individuals to have inconsistent preference through time [3]. In fact, preference reversals, or the tendency to switch from a self-controlled choice to an impulsive one as the time to receiving the impulsive choice approaches, have clear implications for financial decision-making. For example, people often create a budget at the beginning of the month or set a financial goal, but then reverse preference and spend impulsively. Impulsive choice, measured by delay discounting, provides one explanation as to why individuals do not adhere to their financial goals.

Steep delay discounting is related to an array of problematic behaviors [9]. Steeper delay discounting is observed in cigarette smokers [4] and individuals who abuse cocaine [10], methamphetamine [11], heroin [12] and alcohol [13] compared to control participants without these problems [14]. Other difficulties related to steep discounting include problematic Internet use [15], obesity [16], risky sexual behavior [17], and problematic gambling [18]. Also, how steeply someone discounts by delay is related to success in treatment outcomes with individuals that discount less having greater success [19]. Other research shows that discounting of different outcomes is consistent within individuals [20,21] meaning that if an individual steeply discounts money, they are likely to discount other outcomes, such as food, steeply as well. Because delay discounting has been found to be a persistent, trait-like pattern of behavior [9,22] decreases in discounting could potentially reduce risky behaviors in general and improve quality of life across a variety of different domains.

A large body of research has demonstrated that delay discounting can be changed through acute experimental manipulations and more intensive intervention (see Koffarnus, Jarmolowicz, Mueller, & Bickel, 2013 for review) [23]. For example, framing, or how the choice is presented, is an acute manipulation that can alter delay discounting. Several researchers have found that framing the delay of the larger outcome as a specific date reduces delay discounting compared to framing time in calendar units (e.g., weeks, months, years) [24,25, 26]. DeHart and Odum extended this finding by demonstrating that framing the delay in units of days resulted in greater discounted compared to framing time in calendar units [26]. Another framing manipulation that has been shown to change delay discounting is the inclusion of an explicit 0 to the immediate and delayed outcomes [27]. In one example, Radu and colleagues found that framing the choice as some money now and $0 at the delay or $0 now and a larger amount at the delay resulted in less discounting compared to the more common method of not explicitly including the $0 amount [28]. Finally, Johnson, Herrmann, and Johnson found that differential framing of the opportunity cost associated with choosing the delayed outcome changes the discounting of monetary outcomes [29]. As the opportunity costs of choosing the larger outcome increased, participants were more likely to choose the immediate outcome.

Intervening on executive functioning and working memory also alters delay discounting. In a series of studies, Hinson, Jameson and Whitney found that engaging in tasks that tax working memory increased impulsive choice as measured by delay discounting [30]. These results generalized across different preparations including how the tasks were presented and the use of hypothetical and real outcomes for the delay-discounting tasks. A study by Bickel and colleagues [31] further supported the role of working memory in delay discounting. For participants with stimulant abuse problems, completing 4 to 15 sessions of working memory training decreased delay discounting (indicating a decrease in impulsivity). Importantly, no change in delay discounting was reported in a sham-training control group, suggesting that the change in delay discounting was not due to multiple task administrations.

Engaging in episodic future thinking is another process that has been found to reduce delay discounting. Peters and Büchel [32] presented participants with cues related to reported positive future events and asked them to make intertemporal choices. They found that participants discounted less during the trials with the future event cues compared to trials without the cues. Daniel, Stanton, and Epstein extended these in obese and normal weight individuals [33]. Participants in the episodic future thinking group discounted hypothetical delayed outcomes less then controls did and also consumed fewer calories when given the opportunity to freely eat a variety of foods. Episodic future thinking has also been shown to reduce delay discounting in individuals with problematic alcohol drinking. Snider, LaConte and Bickel found that presenting cues related to positive future events reduced delay discounting and initial alcohol consumption in an alcohol purchase task [34].

Interventions involving affective processes may also alter delay discounting. Positive affect induction in extroverted participants increased delay discounting [35], suggesting that emotional states influence impulsive choice. DeSteno and colleagues randomly assigned participants to three mood induction groups: neutral, happy, and grateful [36]. They found that participants in the gratitude condition discounted less than participants in the neutral and happy conditions. In a large survey of research relating to affective processes, Fields and colleagues conducted a meta-analysis to investigate the relationship between stress and delay discounting [37]. They found a moderate to strong relationship between stress and delay discounting with higher levels of stress relating to greater discounting. Importantly, many of the studies included experimentally induced stress. An intervention designed to improve one’s ability to cope with negative affective states has also been shown to reduce delay discounting. Morrison and colleagues found that a brief session of Acceptance and Commitment Therapy (ACT), which was designed to help participants increase willingness to experience discomfort, decreased delay discounting [38].

Finally, financial education may also be a method to decrease delay discounting, but research demonstrating this impact is thus far limited. Meier and Sprenger [39] investigated the relation of opting to receive a brief one-time financial counseling session and performance on a monetary choice task. Participants (already at the facility to receive free tax services) could elect to receive a free financial counseling session that included a free credit report. Meier and Sprenger [39] found that participants that elected to receive the brief financial counseling session were more likely to choose the delayed, larger monetary outcome in the monetary choice task compared to participants that did not elect to receive the counseling session. Although these results do not demonstrate that financial education reduces delay discounting, they do suggest that financial behaviors and delay discounting are related.

Black and Rosen [40] successfully influenced delay discounting by enrolling participants with cocaine abuse problems in a money management outpatient treatment program. The intervention included substance abuse treatment, financial training, as well as restriction of access to income and credit cards through a money manager. Participants who received financial training and money management in addition to substance abuse treatment showed no change in delay discounting but were more likely to remain abstinent throughout the course of treatment. Participants who did not receive the financial training and management (but did receive financial workbooks to complete on their own time) showed increased delay discounting and were more likely to relapse during treatment. Black and Rosen’s [40] study provides evidence that financial education and management buffered against the subsequent increase in delay discounting and relapse as treatment progressed. However, the ability of financial education alone to affect delay discounting in non-substance abusing populations has not been investigated.

Financial education may provide a powerful tool to decrease delay discounting and related impulsive behavior. Further research is needed to establish this effect by itself, without intensive personal money management intervention, and beyond a population with substance abuse disorders. In the present study, we investigated the ability of a semester-long personal finance course to decrease delay discounting in undergraduate college students.

## Methods

The Utah State University Internal Review Board approved this study. All participants completed an informed consent before completing the study. Participants were recruited from an introductory personal finance course (n = 318) and an abnormal psychology course (n = 96). In the analysis and results, the abnormal psychology course participants will be referred to as the control group (described below) and the personal finance course participants will be referred to as the financial education group. The participants received either extra course credit or laboratory credit for participation in the study; alternative sources of credit were offered for non-participants. Instructors from both courses were aware of the study but were not part of the research team.

The personal finance course focused on basic financial education. The topics of focus were: career planning, employment benefit packages, budgeting and budgeting tools, reducing expenses, taxes, family expenses, credit and credit reports, credit cards, loans (e.g., mortgage, installment), debt, interest, housing, property and liability, healthcare, insurance (e.g., car, home, and health), investing (e.g., stocks, bonds, mutual funds), and retirement saving (e.g., IRA, 401k accounts). Personal finance course students also completed several assignments during the semester including tracking their monthly expenses, budgeting, and exploring home ownership. Class sessions for the course were fifty minutes and were held three times a week.

The abnormal psychology course focused on a basic spectrum of psychopathology (psychological disorders). The abnormal psychology course served as a control group because the students in the two courses share similar demographics (e.g., class year and diversity of majors) and both courses are popular general education courses. Students in the abnormal psychology course did not receive financial education. There were no dually enrolled participants.

Participants completed an online multi-part survey at two different times: immediately after the semester began (Time 1) and two weeks prior to the start of final examinations (Time 2). This time constituted a 12-week span between the Time 1 and Time 2 surveys. The survey consisted of demographic questions (age, sex, GPA, and academic major), an online version of a 125-question delay discounting measure [4], a 44-item personality inventory [41], and a five-item financial risk tolerance scale [42]. The second administration of the survey was identical to the first with the exception of the addition of an additional delay discounting assessment at the end of the survey, as explained below.

In the delay-discounting task, participants chose between hypothetical outcomes of a smaller, immediate amount of money and $100 after a delay [5, 43]. The progression of immediate amounts began at $100 and decreased to $1 ($100, $99, $97.50, $95, $92.50, $90, $85, $80, $75, $70, $65, $60, $50, $45, $40, $35, $25, $20, $15, $10, $7.50, $5, $2.50, $1 [29]. The delay to receipt of the larger amount of money increased across five blocks: 1 week, 1 month, 1 year, 5 years, and 25 years. The instructions for each delay block read: “In this section, you will be asked to make a series of choices between hypothetical monetary alternatives. These choices will be displayed on the screen. For each question, one amount of money is to be paid right now, and this amount will vary from question to question. The other amount of money will remain fixed, but its payment will be delayed. The question will inform you how long the delay will be. For each choice, if you would prefer to have the amount that is shown now, select the ‘US$ XX now’ button. If you would prefer to have the amount that is shown for the ‘delay’ (e.g., ‘1 week’), click on the delay button. There are no correct or incorrect choices. You are not expected to choose any particular way. Please just choose the one you would prefer. You will not receive any of the money described here, but it is important that you choose the way you think you would if the money were real.” This delay-discounting task was chosen because it is widely used, well-established, and readily administered using basic survey software.

For each delay block, all 24 amounts were displayed on the screen at once in two columns. For each amount, the participant was prompted to choose between the immediate amount (left column) and the delayed amount (right column). An indifference point was obtained for each delay by identifying the question when the participant switched their preference from the delayed to the immediate option. The repeated assessment of delay discounting, which participants completed at the beginning and end of the semester, asked participants about a delayed monetary gain of $100. The assessment of delay discounting that was only administered during the second survey at the end of the semester asked the participants about a delayed monetary gain of $1000. The immediate amounts were the same as in the $100 discounting task, but the decimal place was moved to the right one position. The addition of the second measure served as a manipulation check because previous research has shown that larger amounts of money are discounted less steeply than smaller amounts [44].

Participants also completed the 44-item personality inventory [41]. This inventory is a ‘Big Five’ personality inventory that assessed a participant’s trait extraversion, agreeableness, conscientiousness, emotional stability, and their openness. Each question consisted of a self-rating on a five-point Likert scale about a specific question, such as “I see myself as someone who is talkative.”

The 5-item Financial Risk Tolerance Scale was used to determine a person’s investment risk tolerance [42]. This four-point Likert-type response scale ranges from 1 (strongly agree) to 4 (strongly disagree). Higher scores indicate higher risk tolerance.

### Analyses

Participant data were examined to ensure that participants completed both tasks and met systematic discounting criteria. Data from a total of 88 participants were removed from the final analyses for two reasons: if participants did not complete both administrations of the task (n = 30) or responded on the delay discounting measure in a unsystematic manner (n = 58) as established by an algorithm for identifying non-systematic data [45]. The first criterion of the algorithm excludes participant data for which an indifference point is larger than the previous indifference point by 20% of the amount of the delayed outcome (in this case $20). The second criterion of the algorithm excludes participant data in which the final indifference point is larger than 90% of the first indifference point. Data that failed either criterion were removed from analyses. Analyses of demographic variables between the full sample and the purged sample revealed no significant differences between the groups. The final analyses included data from 237 financial education participants and 80 abnormal psychology participants.

#### Analysis of delay discounting

The measure reported for the delay-discounting task is Area Under the Curve (AUC) [46]. AUC is bound between 0 and 1, with lower AUC values indicating a higher degree of delay discounting. AUC is the sum of the trapezoidal area between each set of adjacent indifference points and is calculated using the formula (*x*_*2*_ –*x*_*1*_)[(*y*_*1*_ + *y*_*2*_)/2]. x_1_ and *x*_*2*_ are successive delays and *y*_*1*_ and *y*_*2*_ are the indifference points for those delays. A benefit of using AUC as the unit of analysis is that it does not rely on a theoretical model of delay discounting. An increase in AUC reflects a decrease in delay discounting whereas a decrease in AUC reflects an increase in delay discounting.

Generalized Estimating Equation (GEE) modeling (repeated measures regression analysis) was used as the primary analysis [47]. This analysis was chosen because of its robust abilities to handle unequal sample sizes for group comparisons, and to analyze repeated measures data. GEE analyses report two key values. The first is a ß value, which can be interpreted as a standard regression coefficient. The ß value indicates the degree of change in AUC from Time 1 to Time 2 predicted by an independent variable. The Robust *Z* value is a *z*-statistic (similar to a *t*-statistic) that is adjusted for the correlation of the multiple dependent measures. A statistically significant Robust *Z* value indicates that the ß for that parameter is statistically significant. For all pairwise comparisons, a Bonferroni adjustment was applied to account for multiple comparisons. The model was adjusted to account for the inter-correlation of AUC scores at Time 1 and Time 2 by using an unstructured correlation matrix, which is most appropriate when there are few time points [48]. All analyses were conducted using The R Project for Statistical Computing [49]. For the GEE analysis, the geepack package [50] was used for the main GEE analysis and the lsmeans package was used for any additional comparisons [51]. All participant results are available as S1 File.

## Results

The results were first analyzed for two factors: demographic equivalence between the control (mean age = 22.26 years; mean GPA = 3.42; 29 males, 51 females) and financial education (mean age = 22.15 years; mean GPA = 3.45; 91 males, 146 females) groups at Time 1, and AUC equivalence between groups at Time 1 (mean control AUC = 0.45; mean financial education AUC = 0.42). The control and financial education groups were did not differ in age *t*(153.8) = 0.22, p = .82, *d* = 0.03), GPA (*t*(142.49) = -0.62, p = .54, *d* = -0.08) or degree of delay discounting (AUC) at Time 1 (*t*(135.82) = 0.84, p = .40, *d* = 0.11). A Chi-Square test revealed that sex was distributed equally across groups (*X*^*2*^ = .044, N = 317, *p* = .836).

Results from the personality inventory (The Big Five Personality Inventory) were included in the model analysis, but the measures were not consistent across time. For three of the traits, the paired sample *t*-tests demonstrated significant changes in composite scores from Time 1 to Time 2. Participants reported becoming less agreeable (Agreeableness mean Time 1 = 37.08, Time 2 = 36.36; *t*(316) 1.93, *p* = .05, *d* = 0.11) and more open (Openness mean Time 1 = 32.53, Time 2 = 36.08; *t*(316) = -9.092, *p* < .000, *d* = -0.51) at end of the semester than at the beginning of the semester. Participants also reported becoming less conscientious from Time 1 to Time 2 (Conscientiousness mean Time 1 = 35.27, Time 2 = 34.55; *t*(316) = -1.963, p = .05, *d* = 0.11). No differences in Extroversion (mean Time 1 = 26.85, Time 2 = 26.04; *t*(316) = 1.85, p = .07, *d* = 0.10) or Neuroticism (mean Time 1 = 22.09, Time 2 = 22.34; *t*(316) = -0.61, p = 0.54, *d* = -0.03) were found across time. Big Five Personality scores were equal between groups at both times (collapsed across groups, *t*s(143.63) < 1.77, ps < .08). Big Five Personality Inventory scores were only included as independent predictors, not interaction terms with time or group to avoid interpreting three-way interactions.

### Between Group Model

**Fig 1:**
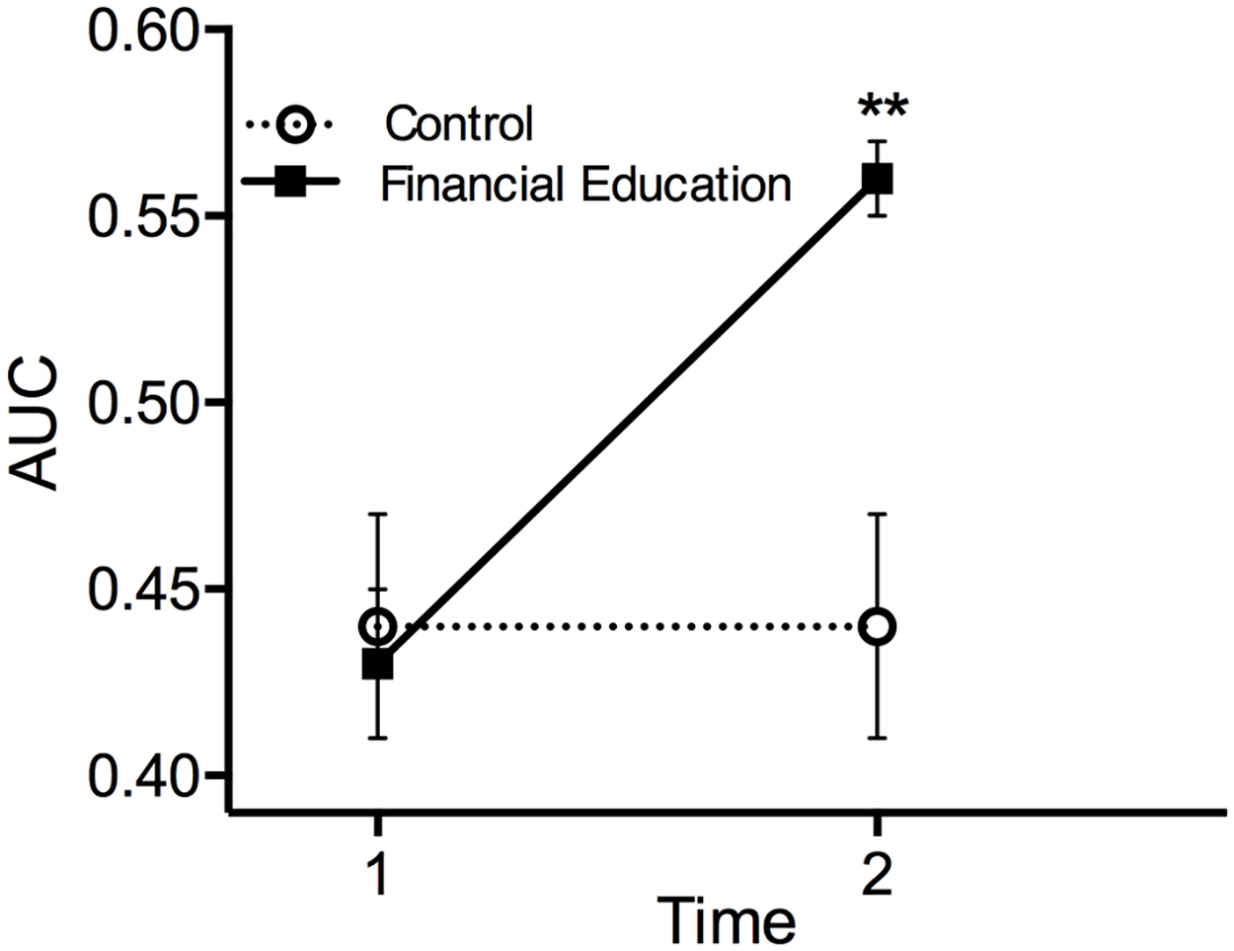
Change in AUC. Change in mean AUC for $100 for the Financial Education (solid line) and Control (dotted line) groups. AUC significantly increased in the financial education group whereas delay discounting did not change for the control group.

To investigate the factor of main interest, effects of the financial education on delay discounting, generalized estimating equation analyses were conducted. AUC for $100 for participants in the financial education group increased (discounting decreased) from Time 1 to Time 2 whereas AUC for $100 for participants in the control group did not change (Fig 1). The final model is reported in Table 1; S1 Table contains the entire model process.

**Table 1:** Between group GEE results.

	ß	Std. Error	Robust Z
Intercept	0.02	0.08	0.30
Time	-0.01	0.05	-0.45
Group	-0.16	0.05	-3.30***
GPA	0.02	0.02	0.78
Sex	0.04	0.02	2.44**
AUC $1,000	0.59	0.03	18.92***
Time x Group	0.12	0.03	4.28***

Dependent variable is change in AUC for $100 from Time 1 to Time 2.

* p < .05,

** p < .01,

*** p < .001.

A statistically significant main effect for group membership (ß = 0.16, p < .01) indicated that AUC was higher overall for the financial education group compared to the control group. There was no significant main effect of time (ß = -0.01). Importantly, a statistically significant interaction of time by group membership (ß = 0.12, p < .001) demonstrates that membership in the financial education group resulted in a statistically significant increase in AUC for $100 (decrease in delay discounting) from Time 1 to Time 2 whereas membership in the control group did not result in an increase in AUC for $100 from Time 1 to Time 2 (Fig 1). AUC for $100 (averaged across time) was higher in women (ß = 0.04, p < .05) compared to AUC for men. However, sex did not interact with time or group membership. Therefore, although AUC for $100 for participants with higher GPA’s and for women were higher than participants with lower GPA’s and for men, these variables did not differentially predict changes in AUC for $100 in the financial education group. AUC for $1,000, measured at Time 2, also significantly predicted AUC for $100 (aggregated across time; ß = 0.59, p < .001). Finally, financial risk tolerance did not predict a change in AUC for $100 and was therefore not included in the final model.

To analyze how delay discounting at different time points and for different amounts was related within an individual, bivariate correlations of AUC were investigated. AUC for $100 at Time 1 was strongly positively correlated with AUC for $100 at Time 2 (*r*(317) = .558, p < .001). AUC for $1000 was also strongly positively correlated with at AUC for $100 at Time 1 (*r*(317) = .581, p < .001 and AUC for $100 at Time 2 (*r*(317) = 0.713, p < .001). Therefore, although AUC for $100 increased for the financial education group, an individual with a relatively high AUC for $100 at Time 1 also had a relatively high AUC for $100 at Time 2.

To analyze change in self-reported risk tolerance, a GEE model was conducted to compare changes in risk tolerance from Time 1 to Time 2 by group. A significant main effect of group membership was found (ß = -2.17, *Z* = -3.13, p < .001). A significant main effect of time was not found (ß = -0.51, *Z* = -1.39, p = 0.16). A significant interaction of time and group membership was found (ß = 1.73, *Z* = 4.04 p < .001) indicating that self-reported risk tolerance increased in the financial education group but not the control group. Analysis of the interaction of group membership by time revealed that risk tolerance increased for participants in the financial education course (*M* = 10.89 at Time 1 and *M* = 12.11 at Time 2; *z* ratio = -5.59, p < .001, *d* = -0.36) but did not change for participants in the control condition (M = 11.34 at Time 1 and *M* = 10.83 at Time 2; *z* ratio = 1.39, p = 0.98, *d* = 0.15).

To verify that participants in this study discounted in a way comparable to delay discounting assessed by other methods, delay discounting results were further analyzed.

First, delay discounting of different amounts were compared. Participants completed a delay-discounting task for $1,000 at Time 2. Participants in the control group discounted $100 more steeply than $1,000 at Time 1 (*t*(79) = -3.17, p < .01, *d* = -0.35) and Time 2 (*t*(79) = -4.06, p < .001, *d* = = 0.45). Participants in the financial education group also discounted $100 more steeply than $1,000 at Time 1 (*t*(236) = -9.39, p < .001, *d* = -0.61) but not at Time 2 (*t*(236) = -1.64, p = 0.10, *d* = -0.11). Second, a commonly used theoretical model of delay discounting was fit to the median group indifference points for control and financial education participants [2]. The hyperbolic model fit all median group indifference points well and the mean R^2^ value for all model fits was 0.94 (all R^2^ values were above 0.91). At Time 1, the rate of discounting for the control and financial education groups was *k =* 0.0006 and 0.0006, respectively. At Time 2, the rate of discounting for the control and financial education groups was *k* = 0.0007 and *k* = 0.0004 respectively. We conclude that our on-line task measured delay discounting in a comparable way to other investigations of delay discounting.

### Financial Education Participants

To better understand changes in AUC found in the financial education group, a separate GEE model for only financial education participants was conducted to investigate how demographics, the Big Five Personality Inventory, and Financial Risk Tolerance scores affected AUC. This model was necessary to avoid three-way interaction terms in the between groups model. Similar to the between groups model, a saturated model was first created and non-significant variables were systematically removed from the model until a significant model was obtained. This process is reported in S2 Table. The final model is reported here (Table 2).

**Table 2:** Financial education group GEE results.

	ß	Std. Error	Robust Z
Intercept	0.27	0.14	1.89*
Time	-0.18	0.08	2.36**
Neuroticism Time 1	-0.01	0.00	-2.22*
Financial Risk Time 2	0.02	0.01	2.14*
Neuroticism Time 1 x Time	0.01	0.00	1.97*
Financial Risk Time 2 x Time	-0.01	0.00	-2.65**

Dependent variable is change in AUC for $100 from Time 1 to Time 2.

* p < .05,

** p < .01,

*** p < .001.

GEE model results (Table 2) reveal that AUC for $100 increased from Time 1 to Time 2 (ß = 0.18, p < .01). Financial risk tolerance at Time 2 (ß = 0.02, p < .01) and neuroticism scores at Time 1 (ß = -0.01, p < .05) were predictive of aggregate AUC for $100 (mean of Time 1 and Time 2). Interaction effects revealed that financial risk tolerance at Time 2 (ß = -0.01, p < .01) and neuroticism at Time 1 (ß = 0.01, p < .05) moderated the change in AUC with higher financial risk tolerance and higher neuroticism scores predicting higher AUC for $100.

Finally, we investigated if AUC for $100 at Time 1 predicted the degree of increase in AUC for $100 at Time 2 using an Oldham’s correlation [52, 53]. Oldham’s correlation can help to determine how scores at Time 1 predict the degree of change at Time 2. For example, do participants with low AUC scores at Time 1 reported the greatest change in AUC at Time 2? Results of the Oldham’s correlation revealed that the level of discounting reported at Time 1 was not predictive of the degree of change in AUC for $100 at Time 2 (*r*_Oldham’s_ = .00, p = .98). Therefore, although AUC for $100 at Time 1 was correlated with AUC for $100 at Time 2, the degree of change in AUC at Time 2 was not predicted by AUC at Time 1.

## Discussion

Participation in a semester long financial education course proved to be an effective method for decreasing delay discounting. Previous research has indicated that financial education and how one discounts delayed outcomes are related [39,40] but this is the first study to show that financial education can reduce delay discounting. We will first discuss the details of our results including replications of previous studies. Next, we will discuss questions regarding delay discounting that remain unanswered by our results. Finally, we will discuss the mechanisms of how financial education may have reduced delay discounting.

Participants in the financial education course discounted less by delay at the end of the semester than at the beginning, whereas delay discounting for the participants in the control course (abnormal psychology), who did not receive financial education, did not change. No specific demographic variable moderated the change in the financial education course group, indicating that the beneficial aspects of the intervention were not limited to a particular sex or level of academic success. However, this finding within the financial education group was moderated by neuroticism at Time 1 and financial risk tolerance at Time 2. That is, more neurotic participants and participants with greater financial risk tolerance showed a greater improvement in delay discounting. The finding that financial education can reduce delay discounting provides further evidence that delay discounting can be reduced.

Our results replicate several well-established findings in the delay discounting literature. First, we replicated the finding that delay discounting, without intervention (i.e., in the control group), is relatively unchanged across moderate periods of time [54,55]. Delay discounting has previously been shown to remain stable across time frames up to one year [54]. Previous research has also demonstrated that delay discounting can be altered using specific interventions such as working memory training. Usually, these changes are measured shortly after the intervention (minutes to 2–3 weeks afterwards) [31, 35, 38]. Our study is important in showing a change in delay discounting over the course of months of treatment.

Second, we replicated the consistent finding that smaller amounts of money are discounted more steeply than larger amounts [56]. Participants discounted $1000 less at Time 2 than $100 at Time 1. This finding in part helps to validate the online data collection method. Importantly, we found in the financial education group that discounting of $100 at Time 2 was similar to discounting of $1000 at Time 2, suggesting that financial education reduced the effects of amount on delay discounting. Differences in delay discounting across different magnitudes have important implications for financial education. Often, individuals with problematic spending behaviors do not impulsively spend large sums of money at one time but instead impulsively spend smaller amounts frequently [57]. Financial education may help to reduce these behaviors. Future research should incorporate a variety of magnitudes at both Time 1 and Time 2 to see if financial education reduces discounting of both large and small outcomes.

Third, we also found that different measures of personality were related to delay discounting and the change in delay discounting in the financial education group. However, the effects of different facets of personality on the change in delay discounting should be interpreted with caution as Big Five Personality Inventory scores changed across the semester. Although it is not uncommon to find differences in personality scores when comparing different age cohorts [58] as well as longitudinal changes within individuals [59] such changes over the course of four months are not expected. Financial Risk Tolerance at Time 2 also predicted the observed decrease in delay discounting at the end of the semester. Participants with greater financial risk tolerance reported less delay discounting at Time 2.

Finally, we replicated previous research findings that how a person discounts one outcome is predictive of how they discount other outcomes [20,21]. AUC for $100 at Time 1 was strongly positively correlated with AUC for $100 at Time 2 for both groups. AUC for $100 at Time 1 and Time 2 was also positively correlated with AUC for $1000. These findings demonstrate that even when delay discounting is altered, participants still discount in a consistent manner. That is, a person who shows relatively steep discounting for delayed money at one time point will tend to show relatively steep discounting for delayed money at another time point. Similarly, a person who shows relatively steep discounting for $100 will also tend to show relatively steep discounting for $1,000. These findings support other evidence that delay discounting may reflect a trait-like tendency in how delayed outcomes are valued [20,21,22]. Research has shown that how an individual discounts one outcome is related to how they discount other outcomes [20,43]. For example, if an individual discounts money steeply, it is likely that they will also discount food or drugs steeply. One important implication of the present study is that intervening on monetary discounting through financial education may generalize to less discounting for other outcomes.

One important and unique component of our intervention is that it was not administered in a controlled setting (e.g., private office in a laboratory). Participants received the intervention by attending a semester long course and engaged the material through assignments and exams. We were unable to control for various extraneous variables such as motivation to engage in the course, attendance, and previous financial knowledge. However, finding an effect despite this lack of strict control suggests that the effects of financial education on delay discounting are robust.

Although we successfully demonstrated that financial education can change delay discounting, several important questions remain unanswered. One question is if the change in delay discounting for the financial education group was long lasting. A follow-up test to determine whether the change in delay discounting persisted longer than the end of the semester would be an important extension.

Another important question is if changing delay discounting results in changes in other behaviors. For example, does a change in monetary delay discounting result in improved financial behaviors such as budgeting and saving? Does changing delay discounting using interventions like working memory training and ACT [31,38], result in actual behavioral change such as reductions in substance abuse? It is possible that participation in the financial education class changed participant behavior regarding how they respond to a financial choice questionnaire but not any actual financial behaviors. The degree of generality to actual target behaviors remains an unanswered question in research on interventions that reduce delay discounting and should be addressed in future research. We also do not know if altering delay discounting of money alters delay discounting of other commodities (e.g., food, health, etc.). Because delay discounting has trait-like qualities, it is possible that affecting monetary discounting would also affect how other outcomes are discounted. Finally, it is unclear how these results would generalize to other populations, such as older adults approaching retirement or individuals in substance abuse treatment facilities. A more personalized financial education program guided by the unique financial needs of the individual may serve as a more effective intervention.

The uncertainty about the relation between changes in delay discounting and behavioral change leads to a more general question regarding the construct of delay discounting as it is currently used. Researchers often refer to delay discounting as an underlying mechanism of many maladaptive behaviors [9, 60]. From this perspective, delay discounting measures a general process that is strongly related to maladaptive behaviors such as cigarette smoking. In this view, delay discounting is the fundamental process that relates to addiction. There is some evidence to support this view. Audrain-McGovern and colleagues, using a longitudinal design, found that steep delay discounting predicted smoking acquisition and that delay discounting did not change as a result of smoking [61]. Similarly, Fernie and colleagues found that steep delay discounting predicted future problematic alcohol use in adolescents [62]. Evidence that steep delay discounting precludes addiction does not exclude the possibility that substance abuse increases delay discounting [63], but there is a growing body of evidence that suggests that steep delay discounting plays a causal role in substance abuse acquisition. Therefore, an intervention that reduces delay discounting would be expected to reduce impulsive choice across behaviors and contexts. If this view of delay discounting is correct, then financial education could have broad impacts on behavior and an individual’s quality of life.

An alternative view is that delay discounting is the aggregate result of various psychological processes that when combined; result in the hyperbolic devaluation of delayed outcomes. For example, a recently proposed additive utility model of delay discounting proposes that delay discounting arises from two distinct processes: the utility of the outcome (itself an aggregate process of amount, hedonic value, etc.) and the disutility of waiting. Importantly, in this model, the parameters that measure the disutility of waiting adjust for the non-linear perception of time. There is ample evidence to suggest that time is not perceived linearly, which may provide one explanation why delayed outcomes are discounted hyperbolically [64]. Theoretical models of delay discounting have included adjustments for this psychological process [6,7].

Additional processes are proposed to underlie delay discounting. For example, research on the relation between working memory [65], numeracy [66], general intelligence [67] and delay discounting suggest that these processes influence the rate at which delayed outcomes are devalued. Any intervention that reduces delay discounting may only be affecting one of these processes. Therefore, observing a change in delay discounting may not result in a change in problematic behaviors because although one of the mechanisms shared by delay discounting and the problem behavior was changed, others may remain intact. Future research should begin to investigate these questions.

Although the exact mechanisms that account for the observed change in delay discounting are unclear, several areas of research provide some guidance. Framing, or the context in which a decision is presented [68], may explain how financial education may alter how delayed outcomes are valued. Some research has shown that framing time as a specific date results in less discounting of delayed hypothetical money compared to time framed in calendar units (e.g., weeks, months, etc.) [69]. One of the components of financial education is developing specific financial goals that include specific dates of meeting those goals. Reframing the delays to financial goals such as purchasing a home or retirement may serve to increase the motivation to obtain those goals.

Financial education may also serve to reduce the perceived risk of saving and investing or to increase an individual’s tolerance for risk. The financial education participants in this study reported an increase in financial risk tolerance compared to the control participants. Delayed outcomes in a delay-discounting task include an implicit assumption of risk, meaning that the delayed outcome is perceived as riskier or less likely to actually be received than the immediate outcome [70]. This explanation is in line with our findings that financial risk tolerance increased over the course of the semester and that financial risk tolerance at Time 2 predicted the change in AUC for $100. Through financial education, individuals may come to better understand the necessity of allowing some risk when saving for retirement and opt for a more delayed outcome.

Finally, financial education was most effective in individuals with higher neuroticism scores. However, the relationship between neuroticism and delay discounting is unclear. Research investigating the relationship between delay discounting and personality traits is limited. Hirsh, Morisano and Peterson [71] found that extraversion predicted higher delay discounting rates but only in individuals with lower cognitive ability. Neuroticism may be indirectly related to delay discounting. Adams and Nettle [72] found that although neuroticism was not directly related to delay discounting, it was significantly related to several measures of time perspective such as the Zimbardo Time Perspective Inventory [73] and how far into the future participants planned their finances. Importantly, measures of time perspective also predicted delay discounting. Therefore, the impact of neuroticism on delay discounting may have been mediated by time perspective. We found that neuroticism was related to delay discounting, even though neuroticism scores did not change over the course of the semester. Although we did not measure time perspective, it is possible that the change in delay discounting actually reflects a change in time perspective over the course of the semester.

This study extends previous work [23,31, 38] demonstrating that delay discounting can be reduced through intervention. Financial education may serve as a cost effective intervention for steep discounting which may in turn reduce costly behaviors such as substance abuse in addition to improving financial behaviors such as saving and budgeting. Further research should seek to understand if financial education causes a lasting change in delay discounting and if these changes generalize to other delayed outcomes.

## Supporting Information

S1 File Participant data. (TXT)

S1 Table Between group GEE model results. Reported values are GEE model fit parameters.
(DOCX)

S2 Table Financial education GEE model results. Reported values are GEE model fit parameters.
(DOCX)
