# Supplementary material for: The Effects of Financial Education on Impulsive Decision Making
Source: PLoS One. 2016 Jul 21;11(7):e0159561. doi: 10.1371/journal.pone.0159561 (PMC4956221; doi:10.1371/journal.pone.0159561)
Supplement: S1 Table — Reported values are GEE model fit parameters. (DOCX) [file pone.0159561.s002.docx]

| **S1 Table. Between group GEE model results.** | | | | | | | | | |
| --- | --- | --- | --- | --- | --- | --- | --- | --- | --- |
|  | Model 1 | Model 2 | Model 3 | Model 4 | Model 5 | Model 6 | Model 7 | Model 8 | **Model 9** |
| Intercept | 0.07 | 0.06 | 0.05 | 0.05 | 0.04 | 0.04 | 0.18 | -0.08 | 0.02 |
| Group | -0.16*** | -0.16*** | -0.16*** | -0.16*** | -0.16*** | -0.16*** | -0.16*** | -0.16*** | -0.16*** |
| Time | -0.01 | -0.01 | -0.01 | -0.01 | -0.01 | -0.01 | -0.01 | -0.01 | -0.01 |
| AUC $1000 | 0.59*** | 0.59*** | 0.59*** | 0.59*** | 0.60*** | 0.60*** | 0.59*** | 0.59*** | 0.59*** |
| GPA | 0.01 | 0.01 | 0.01 | 0.02 | 0.02 | 0.02 | 0.02 | 0.01 | 0.02 |
| Sex | 0.04* | 0.04* | 0.04* | 0.04* | 0.04* | 0.04* | 0.04* | 0.04* | 0.04* |
| Extroversion 1 | 0.00 |  |  |  |  |  | 0.00 |  |  |
| Extraversion 2 | 0.00 |  |  |  |  |  |  | 0.00 |  |
| Agreeableness 1 | 0.00 | 0.00 |  |  |  |  | 0.00 |  |  |
| Agreeableness 2 | 0.00 | 0.00 |  |  |  |  |  | 0.00 |  |
| Contentiousness 1 | 0.00 | 0.00 | 0.00 |  |  |  | 0.00 |  |  |
| Contentiousness 2 | 0.00 | 0.00 | 0.00 |  |  |  |  | 0.00 |  |
| Neuroticism 1 | 0.00 | 0.00 | 0.00 | 0.00 | 0.00 |  | 0.00 |  |  |
| Neuroticism 2 | 0.00 | 0.00 | 0.00 | 0.00 | 0.00 |  |  | 0.00 |  |
| Openness 1 | 0.00 | 0.00 | 0.00 | 0.00 |  |  | 0.00 |  |  |
| Openness 2 | 0.00 | 0.00 | 0.00 | 0.00 |  |  |  | 0.00 |  |
| Financial Risk 1 | 0.00 | 0.00 | 0.00 | 0.00 | 0.00 | 0.00 | 0.00 |  |  |
| Financial Risk 2 | 0.00 | 0.00 | 0.00 | 0.00 | 0.00 | 0.00 |  | 0.00 |  |
| Group x Time | 0.12*** | 0.12*** | 0.12*** | 0.12*** | 0.12*** | 0.12*** | 0.12*** | 0.12*** | 0.12*** |
|  |  |  |  |  |  |  |  |  |  |
| QIC | -2187.83 | -2192.11 | -2196.85 | -2201.54 | -2201.94 | -2206.18 | -2199.02 | -2197.20 | -2209.45 |

Reported values are GEE model fit parameters.

* p < .05, ** p < .01, *** p < .001
