# Supplementary material for: The Effects of Financial Education on Impulsive Decision Making
Source: PLoS One. 2016 Jul 21;11(7):e0159561. doi: 10.1371/journal.pone.0159561 (PMC4956221; doi:10.1371/journal.pone.0159561)
Supplement: S2 Table — Reported values are GEE model fit parameters. (DOCX) [file pone.0159561.s003.docx]

**S2 Table. Financial education GEE model results.**

|  | Model 1 | Model 2 | Model 3 | Model 4 | Model 5 | Model 6 | Model 7 | Model 8 |
| --- | --- | --- | --- | --- | --- | --- | --- | --- |
| Intercept | -0.13 | -0.10 | -0.16 | 0.04 | 0.35 | 0.50 | 0.38* | 0.27* |
| Time | 0.15 | 0.14 | 0.17 | 0.20 | 0.05 | 0.01 | 0.09 | 0.18** |
| Sex | 0.06 | 0.07 | 0.06 | 0.07 | 0.08 |  |  |  |
| GPA | 0.02 |  |  |  |  |  |  |  |
| Extraversion 1 | 0.00 | 0.00 |  |  |  |  |  |  |
| Extraversion 2 | 0.00 | 0.00 |  |  |  |  |  |  |
| Agreeableness 1 | 0.01 | 0.00 | 0.00 |  |  |  |  |  |
| Agreeableness 2 | 0.00 | 0.00 | 0.00 |  |  |  |  |  |
| Contentiousness 1 | 0.00 | 0.00 | 0.00 | 0.00 |  |  |  |  |
| Contentiousness 2 | 0.01 | 0.01 | 0.01 | 0.01 |  |  |  |  |
| Neuroticism 1 | 0.01 | 0.01 | 0.01 | -0.01* | -0.01* | -0.01* | -0.01** | -0.01* |
| Neuroticism 2 | 0.00 | 0.00 | 0.00 | 0.00 | -0.01 | 0.00 |  |  |
| Openness 1 | 0.00 |  |  |  |  |  |  |  |
| Openness 2 | 0.00 |  |  |  |  |  |  |  |
| Financial Risk 1 | 0.01 | -0.01 | -0.01 | -0.01 | -0.01 | -0.01 | -0.01 |  |
| Financial Risk 2 | 0.02 | 0.02 | 0.02 | 0.02* | 0.02* | 0.02 | 0.02* | 0.02* |
| Sex*Time | 0.02 | -0.02 | -0.02 | -0.02 |  |  |  |  |
| GPA*Time | 0.01 |  |  |  |  |  |  |  |
| Extraversion 1*Time | 0.00 | 0.00 |  |  |  |  |  |  |
| Extraversion 2*Time | 0.00 | 0.00 |  |  |  |  |  |  |
| Agreeableness 1*Time | 0.00 | 0.00 | 0.00 |  |  |  |  |  |
| Agreeableness 2*Time | 0.00 | 0.00 | 0.00 |  |  |  |  |  |
| Contentiousness 1*Time | 0.00 | 0.00 | 0.00 | 0.00 |  |  |  |  |
| Contentiousness 2*Time | 0.00 | 0.00 | 0.00 | 0.00 |  |  |  |  |
| Neuroticism 1*Time | 0.00 | 0.00 | 0.00 | 0.00 | 0.00 | 0.00 | 0.01** | 0.01* |
| Neuroticism 2*Time | 0.00 | 0.00 | 0.00 | 0.00 | 0.00 | 0.00 |  |  |
| Openness 1*Time | 0.00 |  |  |  |  |  |  |  |
| Openness 2*Time | 0.00 |  |  |  |  |  |  |  |
| Financial Risk 1*Time | 0.01 | 0.01 | 0.01 | 0.01 | 0.01 | 0.01 | 0.01 |  |
| Financial Risk 2*Time | -0.01 | -0.01 | -0.01 | -.01* | -0.01** | -0.01* | -0.01** | -0.01** |
|  |  |  |  |  |  |  |  |  |
| QIC | -1385.25 | -1389.66 | -1396.34 | -1396.87 | -1392.49 | -1391.58 | -1394.60 | -1397.79 |

Reported values are GEE model fit parameters.

* p < .05, ** p < .01, *** p < .001
